# Organotypic 3D Models of the Ovarian Cancer Tumor Microenvironment

**DOI:** 10.3390/cancers10080265

**Published:** 2018-08-09

**Authors:** Karen M. Watters, Preety Bajwa, Hilary A. Kenny

**Affiliations:** 1 Department of Chemistry, University of Chicago, Chicago, IL 60637, USA; kmwatters@uchicago.edu; 2 Department of Obstetrics and Gynecology, Section of Gynecologic Oncology, University of Chicago, Chicago, IL 60637, USA; preetybjw25@gmail.com

**Keywords:** ovarian cancer, tumor microenvironment, 3D models

## Abstract

Ovarian cancer progression involves multifaceted and variable tumor microenvironments (TMEs), from the in situ carcinoma in the fallopian tube or ovary to dissemination into the peritoneal cavity as single cells or spheroids and attachment to the mesothelial-lined surfaces of the omentum, bowel, and abdominal wall. The TME comprises the tumor vasculature and lymphatics (including endothelial cells and pericytes), in addition to mesothelial cells, fibroblasts, immune cells, adipocytes and extracellular matrix (ECM) proteins. When generating 3D models of the ovarian cancer TME, researchers must incorporate the most relevant stromal components depending on the TME in question (e.g., early or late disease). Such complexity cannot be captured by monolayer 2D culture systems. Moreover, immortalized stromal cell lines, such as mesothelial or fibroblast cell lines, do not always behave the same as primary cells whose response in functional assays may vary from donor to donor; 3D models with primary stromal cells may have more physiological relevance than those using stromal cell lines. In the current review, we discuss the latest developments in organotypic 3D models of the ovarian cancer early metastatic microenvironment. Organotypic culture models comprise two or more interacting cell types from a particular tissue. We focus on organotypic 3D models that include at least one type of primary stromal cell type in an ECM background, such as collagen or fibronectin, plus ovarian cancer cells. We provide an overview of the two most comprehensive current models—a 3D model of the omental mesothelium and a microfluidic model. We describe the cellular and non-cellular components of the models, the incorporation of mechanical forces, and how the models have been adapted and utilized in functional assays. Finally, we review a number of 3D models that do not incorporate primary stromal cells and summarize how integration of current models may be the next essential step in tackling the complexity of the different ovarian cancer TMEs.

## 1. Introduction

From tumor initiation to metastasis, intricate and reciprocal interactions between ovarian cancer cells and the stromal components of their surrounding milieu create complex and fluctuating tumor microenvironments (TMEs) [1,2]. Stromal components of the TME include the tumor vasculature and lymphatics (including endothelial cells and pericytes), mesothelial cells, fibroblasts, immune cells, and extracellular matrix (ECM) proteins. Mechanical forces such as sheer stress caused by increased peritoneal fluid flow also contribute to this environment, inducing changes in cell morphology and gene expression [3,4]. All of these elements are associated with specific facets of tumorigenesis and metastasis, and their involvement cannot be accurately captured by traditional 2D cell culture systems. Cancer cell cultures in 3D microenvironments are far more representative of disease than traditional 2D systems. Three-dimensional systems provide 1) conditions which are structurally similar to the in vivo environment and are amenable to changes in oxygen and growth factor gradients (e.g., cell spheroids) [5] and 2) cell–cell and cell–ECM communication (e.g., scaffold-based models) [6,7].

**Figure 1:**
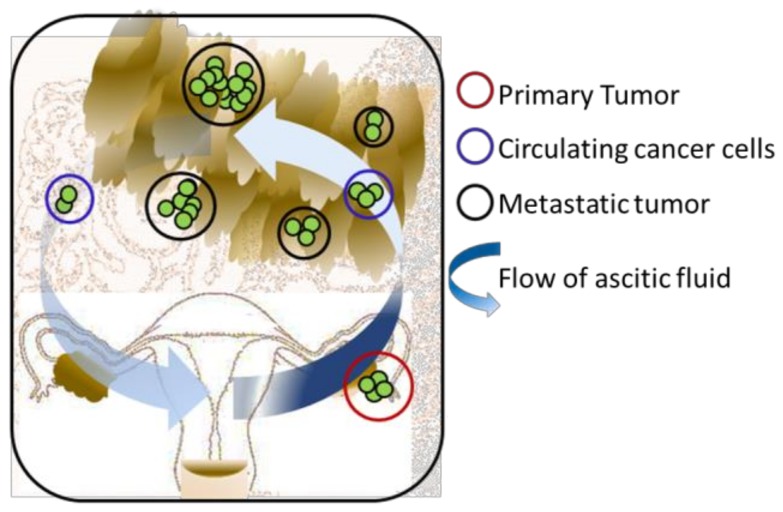
Pathogenesis of the ovarian cancer disease.

Ovarian cancer progression involves detachment of cancer cells from the in situ carcinoma in the fallopian tube or the primary ovarian tumor, dissemination into the peritoneal cavity as single cells or spheroids, and attachment to the mesothelial-lined surfaces of the omentum, bowel, and abdominal wall [8,9] (Figure 1). Ovarian cancer complexity and heterogeneity has meant that development of in vitro 3D ovarian cancer TME models to recapitulate in vivo pathophysiological features has been challenging. Our group has previously published comprehensive reviews of the different 3D culture methods used to study the ovarian cancer TME [10,11,12]. In the current review, we focus on the latest organotypic 3D models that utilize primary stromal cells, in particular a 3D model of the omental mesothelium and a microfluidic model. We provide an overview of these models, both of which are used to study the early steps of ovarian cancer metastasis, describing the cellular and non-cellular components, the consideration of mechanical forces, and their utilization. We discuss the challenges and limitations associated with the current models and put forward the essential steps to establish an archetype model that will faithfully recreate the in vivo scenario. 

## 2. Three-Dimensional Modelling of Early Metastasis TME Interactions in Ovarian Cancer

Organotypic models refer to 3D models, usually containing ECM, that are comprised of two or more cell types to mimic the complex interactions within a tissue. For this review, we focus on organotypic models of the ovarian cancer TME that comprise a 3D culture containing at least one primary stromal cell type in an ECM background, such as collagen or fibronectin, plus ovarian cancer cells. 

Ovarian cancer cells have a special predilection for the peritoneum and the omentum as sites of metastasis [8]. The outer lining of these sites consists of a single layer of mesothelial cells with an underlying ECM. During the metastatic process, microscopic non-invasive omental metastases proliferate on top of this layer of mesothelial cells. As the metastases increase in size, the cancer cells induce pro-tumorigenic changes in the stromal cells of the microenvironment, including an increase in the number of fibroblasts and a more rigid basement membrane [13]. Tumor cells then invade the omental adipose tissue. In 1985, Niedbala et al. were the first to establish an organotypic culture of the ovarian cancer TME and investigate the mechanism through which ovarian cancer cells infiltrate the mesothelial cell layer and attach to the ECM [14]. Human primary mesothelial cells (HPMCs) were grown in a monolayer on ECM derived from bovine corneal endothelial cells, onto which ovarian cancer cells derived from patient ascites were seeded. A current version of this organotypic model of the ovarian cancer TME was developed by Kenny et al. This model allows examination of the role that the ECM, HPMCs and fibroblasts play in the initial adhesion, migration, invasion and proliferation of ovarian cancer cells during early metastasis to the mesothelium [13]; it is referred to in this review as the “mesothelium model”. Adding a different element, other models recreate the dynamic mechanical forces that act upon ovarian cancer spheroids in the peritoneal cavity using microfluidic devices [15,16].

## 3. Three-Dimensional Organotypic Model of Human Mesothelium

**Figure 2:**
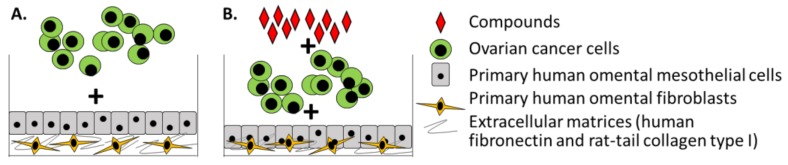
Three-dimensional organotypic model of human mesothelium. (**A**) Layered model for investigating ovarian cancer adhesion, migration, invasion and proliferation in a metastatic microenvironment. In this layered model, the extracellular matrix (ECM) and fibroblasts are cultured together prior to the sequential addition of mesothelial cells and cancer cells. (**B**) Model for high-throughput screening (HTS) to identify compounds that inhibit ovarian cancer adhesion/invasion or proliferation. In this HTS model, ECM, fibroblasts and mesothelial cells are plated simultaneously, followed by the addition of cancer cells and compounds.

The 3D organotypic mesothelium model was created to elucidate the role of specific cellular and non-cellular components, namely fibroblasts, HPMCs, and different ECM proteins, in early ovarian cancer metastasis to the omentum [13]. Two key factors set this mesothelium model apart from other 3D cultures: (1) prior to construction of the model, the authors analyzed hematoxylin and eosin (H&E) stains of normal omental biopsies to form the best picture of the physiological framework of normal omentum and (2) the authors included two types of primary stromal cells, HPMCs and fibroblasts. Using primary HPMCs and fibroblasts at early passages extracted from fresh biopsies of omentum obtained during surgery, the authors recreated the omental ovarian cancer TME in vitro (Figure 2A). Primary human omental fibroblasts were embedded in ECM and overlaid with a layer of HPMCs (1:5 ratio of fibroblasts and HPMCs). With the addition of ovarian cancer cells or immortalized ovarian surface epithelial cells, this highly reproducible construction was used to determine the role of each of the TME components, including different ECM proteins, during ovarian cancer adhesion and invasion. Results from the model showed that both HPMCs and fibroblasts play key roles in these processes. Customization of the model with different ECM proteins revealed that ovarian cancer cell adhesion and invasion is greatest in the presence of collagen, compared with vitronectin, fibronectin, or laminin. This tool was also shared as a JoVE video article to improve dissemination of the protocol and as a resource for other scientists in the ovarian cancer field [17]. 

This modular mesothelium model has been used in numerous publications that further illuminate the mechanisms involved in early ovarian cancer metastasis [18,19,20,21,22,23,24]. Kenny and colleagues proceeded to show that ovarian cancer cells recruit HPMCs to establish metastatic colonies by inducing an upregulation in the levels of fibronectin 1 (FN1) mRNA and protein in HPMCs which promote cell adhesion [18]. They also demonstrated that adhering ovarian cancer cells express matrix-metalloproteinase, which cleaves matrix proteins into smaller fragments, thereby facilitating invasion [19,20]. Other functional assays using the model and an antibody against the urokinase plasminogen activator (uPA) receptor (u-PAR) revealed that targeting the uPA/u-PAR proteolytic system reduced metastasis and induced apoptosis of ovarian cancer cells [21]. Building on this, Mitra et al. used the mesothelium model to identify miRNAs involved in omental colonization, demonstrating that upregulation of uPA in ovarian cancer cells is due to downregulation in miR-193b levels, which is in turn due to ovarian cancer cell interaction with HPMCs on the surface of the omentum [22]. More recently, a study by Caroline Ford’s group used this model to expand on the synergistic role of Wnt receptors ROR1 and ROR2 in early ovarian cancer metastasis, specifically their role in ovarian cancer cell adhesion to the omentum [23].

The ability to customize the mesothelium model led to its reshaping and utilization in high throughput screening (HTS) assays. Through optimization of parameters such as incubation time, plating sequence, number of ovarian cancer cells, HPMCs, fibroblasts, and ECM, the model was adapted for use in reproducible 384- and 1536- multi-well HTS assays [25] (Figure 2B). Fully automated 3D HTS assays were carried out to screen small molecule inhibitors that could potentially target ovarian cancer adhesion/invasion or proliferation [25,26]. The effect of oncology drugs from three small molecule compound libraries, the National Center for Advancing Translational Sciences (NCATS) Mechanism Interrogation PlatE oncology collection, the Prestwick library, and the Library of Pharmacologically Active Compounds (LOPAC^1280^) on ovarian cancer adhesion/invasion or proliferation was investigated. These assays were followed by confirmatory, counter, and secondary biological assays utilizing the 3D organotypic model of human mesothelium to identify lead compounds. Ultimately, inhibitory activity of the lead compounds on ovarian cancer metastasis was validated in different in vivo xenograft models. A key takeaway from the HTS assays was that many of the compounds screened were active in cancer cells on plastic (>90%), but only a few compounds were effective in the 3D HTS platform (<1%) which directly translated to in vivo activity in xenograft mouse models. Differences in drug response between 2D cultures and 3D organotypic models have also been demonstrated in studies of skin melanoma. These studies reported that treatment with tumor necrosis factor-related apoptosis-inducing ligand (TRAIL) in combination with either UVB or cisplatin killed melanoma cells in 2D cultures, but only the TRAIL plus cisplatin combination was effective in their layered 3D organotypic skin melanoma spheroid model [27,28,29]. These differences further highlight the value of 3D organotypic models that can accurately represent the complexity of the ovarian cancer TME.

This mesothelium model is a first step at recapitulating the metastatic microenvironment of ovarian cancer, but it still lacks other in vivo factors such as vasculature, adipocytes, and host immune cells. However, it represents a significantly more complex experimental system than ovarian cancer cells grown in monolayer to analyze the complex mechanisms of tumorigenesis and to potentially identify new therapeutics. Omental cells from different patients in the 3D organotypic cultures reveal a broader picture of donor-to-donor variability in terms of drug response, cellular function and cell signaling. 

## 4. Three-Dimensional Organotypic Model of Cancer Cells Circulating in Ascites

**Figure 3:**
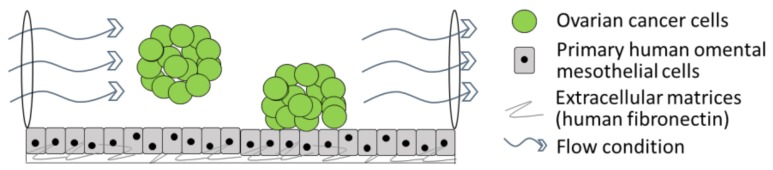
Three-dimensional organotypic model of cancer cells circulating in ascites.

Peritoneal dissemination of ovarian cancer spheroids and their interactions with omental mesothelial cells are not static processes. Hydrodynamic forces generated by increased production of fluid in the peritoneal cavity must be considered in addition to the 3D culture itself. To recreate this aspect of the ovarian cancer TME, Li et al. developed a 3D microfluidic-based platform in which living cells are infused into micrometer-sized chambers [15]. These platforms enable accurate control of the cellular microenvironment, allowing a continuous release of growth factors or nutrients. In their device, Li et al. plated mesothelial cells on fibronectin, and added fluorescently labelled ovarian cancer spheroids under continuous fluidic flow to mimic the flow of peritoneal fluid induced by ovarian cancer in the clinical setting (Figure 3). 

A 2018 publication by Carroll et al. added another layer of complexity by investigating the interactions between alternatively activated macrophages (AAMs), mesothelial cells, and ovarian cancer cells in dynamic flow experiments of ovarian cancer cell adhesion [16]. The authors first determined, under static 3D conditions, that AAM-secreted macrophage inflammatory protein-1 induced expression of P-selectin in mesothelial cell lines, which in turn increased ovarian cancer cell adhesion to the mesothelial cells. Using a parallel-plate flow chamber, which simulates fluid sheer stress on cells, the authors went on to demonstrate that these increased levels of P-selectin in mesothelial cell lines led to increased rolling of ovarian cancer cells. 

Compared with experiments under static conditions, experiments performed under flow conditions provided valuable insights into features of transcoelomic metastasis that cannot be reproduced in standard static cultures, such as increased adhesion under flow conditions [16]. Although these microfluidic 3D models contained only one stromal cell type, their modularity means that they can be customized to include other stromal cells or ECM components for use in functional assays such as adhesion, invasion, and proliferation.

## 5. Other 3D Models of the Ovarian Cancer TME

### 5.1. Organoids

Identification of precursor lesions in the fallopian tube fimbria of ovarian cancer patients and BRCA mutation carriers point towards the fimbria as the likely site of origin of high-grade serous ovarian cancer [30,31,32], but the fallopian tube TME has not been well explored. Organoids are in vitro cellular clusters (3D) derived from primary tissue that use ECM hydrogels to self-assemble with architecture, histology, and genetic features resembling the original tissue [33]. Kessler et al. re-constructed the microenvironmental milieu with growth factors and Matrigel to successfully culture fallopian tube organoids from fallopian tube epithelial stem cells [34]. By supplementing this culture with a selection of growth factors, the authors determined that both Notch and Wnt regulate stemness and differentiation in fallopian tube organoids. 

Fallopian tube organoid in vitro models have also been generated from induced pluripotent stem cells (iPSCs). Yucer et al. guided differentiation of iPSC lines into fallopian tube epithelium precursor cells through exposure to BMP4 and WNT4 followed by follistatin, an activin-binding protein that bio-neutralizes members of the TGF-β superfamily [35]. When spheroids of these differentiated cells were grown on Matrigel and supplemented with estrogen, progesterone, and crucially, conditioned media from primary fallopian tube epithelial cells, they self-organized into luminal structures representative of the fallopian tube architecture with ciliated and secretory components.

Organoids do not contain any stromal components, but can be incorporated into organotypic culture systems to study the interactions between the organoid cells and the cells of their microenvironment. Taking this concept a step further, one could envision a model in which transformed fallopian tube epithelial cells [36] are propagated in organoids and integrated into an organotypic model to investigate the early ovarian cancer TME.

### 5.2. Explant Cultures

While not a model in the sense that models are constructed, explants of omentum, ovary or fallopian tube pieces cultured in the presence of ovarian cancer cells represent another form of 3D culture. In particular, mouse omentum, ovarian and fallopian tube organ pieces can be cultured for up to two weeks [37,38,39]. Human omentum and fallopian tube explants have been cultured for up to five days with ovarian cancer cells [19,40], and ultimately revealed that ovarian cancer cells could metastasize to the fallopian tube. In addition, these explant cultures can be used to test the effect of different drugs or treatments on ovarian cancer adhesion, migration, invasion and proliferation by targeting either the cancer or stromal cells.

### 5.3. Cell Line Spheroids

For most researchers studying the ovarian cancer TME, access to patient tissue to obtain primary cells will be the limiting factor. A number of valuable 3D models that do not include primary cells have been published and utilized in functional assays. These non-organotypic endeavors to recapitulate the primary ovarian cancer TME in vitro include ovarian cancer cell spheroid cultures on synthetic matrices [41], on ECM [9], in low-adherent plastics, in hanging-drops, or in spinner flasks [42,43,44]. While these 3D systems lack a primary stromal cell component, their multi-component concept is more faithful to the TME than cells grown in a monolayer on plastic. Utilization of such systems in numerous studies elucidating the mechanisms of drug resistance demonstrate that they can be used as predictive preclinical models [41,45,46,47,48].

## 6. Challenges and Future Perspectives

### 6.1. Picturing the Prototype Ovarian Cancer TME Model

Developing an ideal model for the ovarian cancer TME is not straightforward. Multiple TMEs with varying components mean that a minimum of four models are likely required: in situ carcinoma in the fallopian tube; dissemination in the peritoneal cavity; early metastatic attachment to the mesothelial-lined surfaces of the omentum, bowel, and abdominal wall; and late chemoresistant metastases. Each complete model will first require the comprehensive characterization (e.g., by immunohistochemistry) of the associated stromal cells and ECM components, the growth factor and metabolite milieu, and, if applicable, the flow rate. Once the components of each TME have been characterized, the primary cells and ECM will need to be isolated, followed by reconstruction of the tissue of interest, with the aid of a bioprinter or synthetic matrices that can be degraded by cells once they form their own ECM architecture. Functionality of the model will then have to be verified. One option for this may be to confirm that the in vitro secreted proteins are analogous to those of the in vivo secretome, for example in terms of drug response or activation of immune cells. Each of these phases of model development is a significant undertaking, and the current models do not come close to the in vivo scenario in terms of the variety of cell types that are involved in each TME.

### 6.2. Future Directions

Multiple potential sites of origin and the continuously changing microenvironments at each stage of the disease demand the development of more diverse (i.e., fallopian tube, ovary, peritoneum) and complex 3D models of the ovarian cancer TME. Each phase of progression has a distinct TME with specific components; for example, models of chemoresistance would include cancer-associated fibroblasts [49], which are not included in the models of early metastasis discussed here. Each of the models presented has its own advantages and limitations, leading us to propose that integration of these models will be a first step towards a more accurate model. 

Currently, the mesothelium model is the only 3D organotypic model of the ovarian cancer TME that is utilized by multiple research groups [13]. The mesothelium model was designed to mimic the tissue organization of the mesothelium that lines the human omentum and peritoneum. It recapitulates the initial adhesion, migration, invasion and proliferation of ovarian cancer cells on the mesothelium lining. This platform has been modified to investigate the individual and cooperative role of different cell types in the TME on ovarian cancer progression. It has evolved and been adapted for HTS of over 100,000 small molecule compounds which could potentially identify new therapeutics for prevention of ovarian cancer metastasis.

The organotypic models discussed here are restrained from reaching their full potential due to the limitations of working with primary tissue, including access to the tissue and the lifespan of the 3D models, the absence of other essential primary features such as vasculature [50,51], and the inclusion of artificial ECM components. Vascularization appears to be next obvious step in advancing the organotypic models towards the in vivo scenario. An elegant model of a vascularized TME was recently published by Magdeldin et al., in which the authors created a 3D model of the tumor stroma using colorectal cancer cell spheroids, collagen hydrogels, the basement membrane protein laminin, human dermal fibroblasts, and human umbilical vein endothelial cells (HUVECs) [52]. Customization of the stromal composition revealed that laminin was critical for regulating vascular network formation, while the addition of the cancer cells to the model disrupted the interconnectivity of the network. Jeon et al. reported on an organ-specific 3D microfluidic model to study human breast cancer cell extravasation during metastasis [53]. In their microfluidic model, primary bone marrow-derived mesenchymal stem cells (hBM-MSCs), osteo-differentiated primary hBM-MSCs, and primary GFP-HUVECs were embedded in a fibrin gel in the microfluidic device. The endothelial cells formed the vasculature, and the other cells contributed to a microenvironment that mimicked bone, a frequent site of metastasis in advanced breast cancer. Addition of breast cancer cells to this modular model enabled the authors to investigate the roles of the different components in extravasation.

Matrices incorporated into the models presented here are purified from other human, rat, or mouse sources. Scaffold properties [54], including the concentration of ECM proteins, can affect the stiffness of the artificial matrix; therefore, the accessibility of drugs in in vitro screening must also be considered and optimized. Incorporation of perictyes and endothelial cells, as well as ECM from patient-matched mesothelium or prolonged cultures where the microenvironmental cells secrete and organize their own ECM, could clarify key mechanisms of metastasis, chemoresistance and recurrence. Bioprinting has emerged as a very promising approach to in vitro 3D cancer models owing to its ability to create complex 3D architectures [55].

Ultimately, 3D organotypic models of ovarian cancer aim to recapitulate but systematically simplify the in vivo human microenvironment. Our hope is that by increasing the physiological relevance of 3D organotypic microenvironment models of tumor initiation, primary tumor growth, circulating tumor multi-cellular aggregates, different metastatic sites, and chemoresistant ovarian cancer, the clinical significance of ovarian cancer research will be improved. If we want to offer personalized medicine for ovarian cancer patients, we will also need to successfully establish ovarian cancer organoids for biobanking, as observed with the establishment of organoid cultures in breast, bladder and colorectal cancers [56,57,58]. By recreating the different TMEs in vitro, we can clarify the role of the TME in the transformation of the original epithelial stem cells into metastatic and chemoresistant cancer cells to ultimately prevent and effectively treat ovarian cancer.
